# Shape Memory PLA/TPU Blend Using High-Speed Thermo-Kinetic Mixing

**DOI:** 10.1021/acsomega.4c04338

**Published:** 2024-12-27

**Authors:** Mona Nejatpour, Ali Fallah, Bahattin Koc

**Affiliations:** † Integrated Manufacturing Technologies Research and Application Center, Sabanci University, Tuzla, Istanbul 34956, Turkey; ‡ Faculty of Engineering and Natural Sciences, Sabanci University, Tuzla, Istanbul 34956, Turkey

## Abstract

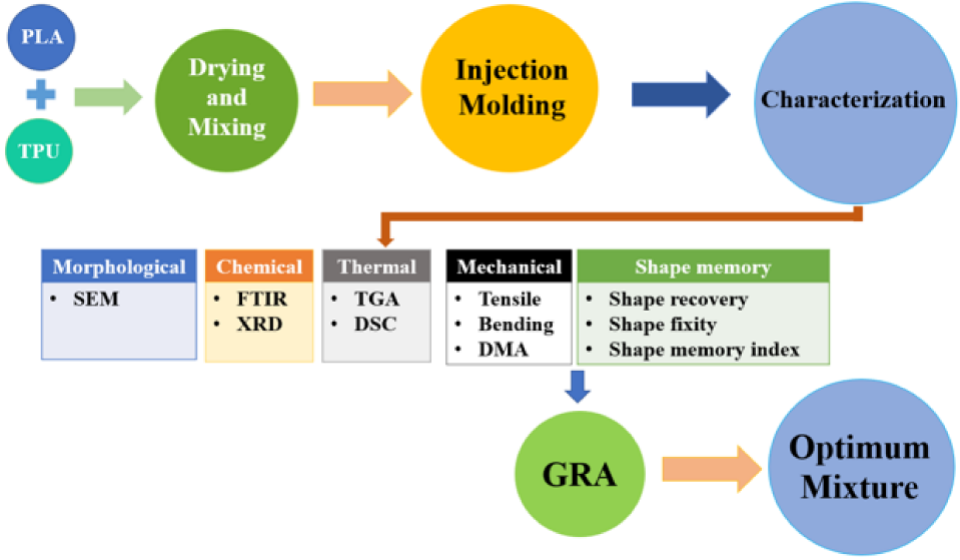

In this study, a thorough examination of the chemical, thermal, and mechanical characteristics, as well as shape memory behavior at low temperatures, of blends consisting of polylactic acid (PLA) and polyurethane (TPU) is conducted. The research involves the preparation of PLA/TPU mixtures with varying concentrations of TPU using a high-speed thermo-kinetic mixing approach. Chemical, morphological, and thermal analyses were conducted on pure PLA, TPU, and PLA/TPU mixtures by using Fourier Transform Infrared (FTIR), X-ray diffraction pattern spectroscopy (XRD), scanning electron microscopy (SEM), thermogravimetric analysis (TGA), differential scanning calorimetry (DSC), and dynamic mechanical analysis (DMA). Mechanical properties were assessed through tensile and three-point bending tests. The achievement of a uniform mixture is confirmed through SEM images, reduction in the glass transition temperature according to DSC and DMA, and an improvement in mechanical properties compared to results documented in the literature, implying a more effective mixing method for the compounds. To assess the practical applicability of this blend, an investigation into the shape memory properties of the mixture when deformed at low temperatures, i.e., cold programming) is carried out. Gray relational analysis (GRA) is employed to identify the optimal TPU content for the mixture, considering both mechanical and shape memory properties. The results indicate that a mixture with a 20% volume fraction of TPU exhibits mechanical properties comparable to those of pure PLA, along with sufficient flexibility at room temperature and notable shape recovery properties.

## 1 Introduction

Shape memory materials (SMMs) are smart materials that can stay in a deformed shape and return to their permanent shape upon exposure to various external stimuli, such as heat, light, moisture, pH changes, or electric and magnetic fields.^1^ Among SMMs, thermally induced shape memory polymers (SMPs) are particularly noteworthy due to their unique properties, including high deformability, lightness, affordability, biocompatibility, and printability.^2−6^ The shape memory behavior of SMPs is a result of their molecular and morphological structure as well as their deformation history. The shape-memory cycle of SMPs consists of programming and recovery stages. During the programming stage, the material is deformed from its original shape to a new temporary shape known as the programmed shape. Then, upon applying the external stimuli, SMP will recover its original shape, i.e., the recovery process.^7^ If the programming process takes place below the switching temperature (*T*_SW_) or transition temperature (*T*_trans_), it is known as cold programming (CP), whereas if it occurs above the *T*_SW_, it is called hot programming (HP).^8^ The *T*_SW_ can be either the glass transition temperature (*T*_g_) or the melting temperature (*T*_m_) of the SMP.^9,10^ Once programmed, if the SMP is heated beyond the *T*_SW_ range, it will revert to its original shape, a process referred to as recovery.^10^ HP-based structures require precise environmental control for programming, with the desired shape typically being predetermined. In practical scenarios, meeting these requirements may be impractical for load-bearing components, given the challenges in environmental control and prediction of deformations from applied loads. Conversely, structures based on CP, programmed at temperatures below the *T*_trans_, streamline the programming process, requiring less energy and time. This renders CP-based structures more cost-effective compared to their high-temperature counterparts, making them particularly appealing for industrial-scale applications.^3,10,11^ Among all the available SMPs, PLA-based SMPCs have been investigated widely in recent decades because of PLA biocompatibility, biodegradability, transparency, processability, commercial availability and affordability, high strength, and printability.^12,13^ Pure PLA’s brittleness and low toughness require enhancement for better flexibility and ductility, achieved through blending with biocompatible shape memory polymers like TPU, PA, and PCL known for their biocompatibility and strong mechanical and shape memory characteristics.^14−20^

In the literature, comprehensive research on the shape memory behavior of PLA/TPU SMPCs via CP is limited in comparison with HP. Recently, Rahmatabadi et al.^21^ investigated the HP shape memory behavior of 3D-printed PLA/TPU (PLA granules, poly ester based 90A TPU) structures with a 70/30 ratio. Their study explored the impact of printing parameters on shape memory behavior, employing the Box–Behnken design (BBD) to model the relationships between variables and responses, and reported the shape fixity and shape recovery in the ranges of 58–100% and 53–91%. In another study by Rahmatabadi et al.,^17^ they examined warm programming and HP behavior of 4D-printed commercial PLA/TPU (poly ester based 90A TPU) SMPCs (programmed at 70 and 90 °C) with 10, 30, and 50% TPU. Results showed shape recovery and fixity ratios ranging from 90.9 to 96.4% and 73.2 to 93.2%, respectively. Jing et al.^22^ investigated the shape memory characteristics of 1-mm thin rectangular PLA (Natureworks LLC, 8052D)/TPU (*T*_g_ = −38 °C, Elastollan TPU, 1185A) blends with ratios of 80/20, 70/30, and 60/40 through cold programming at room temperature over three cycles. Their findings reveal immiscibility in the mixtures, and the shape memory and mechanical properties of the blends vary with changes in the TPU content. Notably, high shape recovery is observed for the PLA/TPU blends with 80/20 and 70/30 ratios after deformation at room temperature (RT). It is important to highlight that although shape memory behavior in extremely thin samples has been documented, its practicality for real-world industrial applications may be limited. Lai et al.^23^ examined the shape memory properties of thin PLA (4032 D)/TPU (Desmopan, KU2-8785) thin sheets under both hot, warm, and cold programming conditions, resulting in a notable shape recovery for CP at room temperature at shape recovery temperature higher than 100 °C. Meanwhile shape memory tests were conducted on samples that deviate significantly from realistic application conditions. Sun et al.^24^ investigated the shape memory behavior of a PLA (4032D, pellet form)/TPU (Desmopan 385E) thin films with varying levels of montmorillonite (MMT) nano clay (Cloisite 15A, density of 1.66 g/cm^3^) as a filler, achieving shape recovery >80% for PLA/TPU with 80/20 ratio via cold programming.

The immiscibility of PLA and TPU SMPC is a well-known challenge arising from different chemical structures and properties, making it difficult for them to form a homogeneous mixture. The immiscibility issue can lead to poor mechanical properties, reduced thermal stability, and limited control over the shape memory behavior. Researchers have been exploring various strategies, including compatibilization and novel mixing techniques, to address this immiscibility and unlock the potential of PLA/TPU blends for a wide range of applications.^21,25−28^ Cai et al.,^3^ by introducing PEG (*M*_w_: 2000) as a plasticizer to the PLA (4032D, pellet)/TPU (Desmopan 385E) blend (80/20 ratio) and decreasing the *T*_trans_, studied the cold-programmed shape memory behavior of the blends. Results showed that the addition of 5 and 10 wt % PEG as a plasticizer enhanced the shape recovery, shape fixity ratio, and shape recovery speed of the SMPCs while increasing the miscibility between PLA and TPU.

The melt blending method in the preparation of SMPCs is a cost-effective, practical, and eco-friendly process since solvents are not used for blending the components.^24,29^ While previous studies commonly used twin-screw extrusion, which has drawbacks like prolonged cycle times and limited dispersion, the Gelimat mixer with high-speed thermo-kinetic mixing has emerged as a superior alternative. This internal mixer overcomes the limitations of twin-screw extrusion, achieving high dispersion in a single cycle due to its high shear rates.^30−32^ In this method, mixing components are accelerated by the blades on a high-speed shaft and followingly generate a large amount of kinetic energy to the particles, which is converted to thermal energy when the particles collide with the chamber wall. The mixing of compounds most likely takes place between the tip of the blade and the wall, mainly because of the centrifugal forces generated by the rotating blade. According to the literature, the dimensions of the Gelimat mixing chamber/shaft and the rotational speed generate high shear rates (about 104 s^–1^) between the tip of the blade and the walls in this system.^30,32,33^ It should be noted that in this method, the mixing time is generally within a few minutes and depends on the charge size, the rotor speed, and the properties of the material used. In this study, the ultrahigh-speed thermo-kinetic mixing method is chosen for its advantages, providing a more efficient solution for developing homogeneous PLA/TPU blends in different ratios with enhanced mechanical properties compared to the related works in the literature related to PLA/TPU SMPCs. Comprehensive characterization of the thermal, chemical, mechanical, and shape memory properties of the PLA mixtures with different amounts of TPU was carried out. A multiresponsive optimization method based on the gray relational analysis was done to find the mixture with the best mechanical and shape memory properties.

## 2 Materials and Method

### 2.1 Materials

Poly lactic acid (PLA 4043 D, Resinex RXP 7502 Natural) and thermoplastic polyurethanes (TPU A72, Ravathane-AG 175 A72 001) were used without further purification.

### 2.2 SMP Mixture Preparation

A Gelimat G1 laboratory-scale high-shear thermo-kinetic mixer (Draiswerke, USA) was used to prepare PLA/TPU mixtures with different TPU content values. To produce the PLA/TPU mixture, both PLA and TPU were first dried in a vacuum oven at 50 °C overnight. All batches were prepared with an identical load of 100 g. To make the mixture homogeneous, the components were first premixed at a low speed (1000 rpm) for 120 s. The mixer was then operated at a shaft speed of 3500 rpm until the blend temperature reached 200 °C, which took between 40 and 60 s. PLA-based SMP mixtures with different contents of TPU (weight percent, wt %) were prepared in about 3 min. The code PLA/TPU-X was used in the sample notation, where X represents the wt % content of TPU in the mixture.

**Figure 1:**
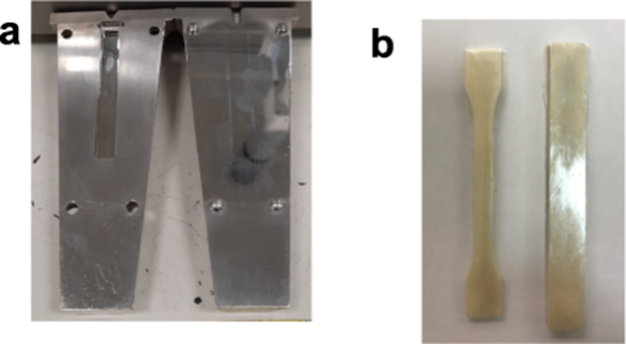
(a) Employed molds for bending samples and (b) final injection molded samples.

The mixtures were then granulated with a typical lab-scale granulator under ambient conditions and, subsequently, injection molded using an Xplore Instruments 12 mL injection molding machine. The injection molding process involved a temperature of 190 °C, an injection pressure of 10 bar, and a mold temperature set at 50 °C. The entire cycle, from injection to completion, took a total of 10 s. Rectangular samples were molded using the ISO 178 standard for three-point bending tests and regular dog bone-shaped samples using the ISO527-2 standard for tensile testing. Rectangular and dog bone molds and final injection molded samples are shown in Figure 1a,b

### 2.3 Characterization Methods

After the PLA/TPU mixture was prepared with different amounts of TPU, a comprehensive characterization of the samples’ chemical, thermal, mechanical, and shape memory properties is done. To this end, SEM imaging, FTIR, XRD, TGA), DSC, DMA, tensile tests, bending tests, and shape recovery tests have been done. The details of each test procedure and conditions are further explained in the following section.

- The phase morphology of SMP blends was inspected by Zeiss LEO Supra 35VP SEMFEG SEM under an accelerating voltage of 10 kV. SEM images were used to analyze the microstructure and morphological changes in polymer composites.
- FTIR measurements were performed using a Nicolet iS10 from Thermo Scientific, covering the infrared spectrum between 600 and 4000 cm^–1^; in transmission mode. The measurements were conducted under nitrogen conditions at a resolution of 2 cm^–1^, with each measurement consisting of 64 scans repeated three times. FTIR analysis helps in identifying new functional groups and understanding the interaction types between PLA and TPU.
- XRD was conducted using a BRUKER Benchtop X-ray Powder Diffraction instrument. The 2-theta X-ray angle was set from 5 to 90° at a rate of 0.02 s ^–1^. The cubic samples, measuring 10 × 10 × 5 mm^3^, cut from the injection-molded samples, were utilized for XRD measurements. XRD results give insight into the impact of adding TPU on the crystalline phases of PLA.
- TGA analysis was carried out using the Shimadzu instrument at temperatures between 25 and 600 °C with a heating rate of 10 °C/min under a nitrogen atmosphere with a 100 mL/min flow rate. Hermetic TA alumina crucibles were used as sample holders with 10–12 mg of granulated samples. TGA results give information about the mixture’s starting and final degradation temperatures.
- DSC analyses were performed using a TA Q2000 instrument calibrated with an indium standard and equipped with Tzero functionality. All measurements were performed between −50 and 180 °C at a heating and cooling rate of 10 °C/min under a nitrogen atmosphere. We applied a typical procedure for sample preparation. About 10 mg of pristine PLA, TPU, and granulated PLA/TPU mixtures were encapsulated in a standard DSC pan prior to measurement. DSC testing is one of the standard methods for determining polymers glass transition temperature (*T*_g_) and melting temperature (*T*_m_).^5^ Moreover, the compatibility of the SMP mixture can be judged by observing a shift in the *T*_g_ of the phases compared to the base polymers. The degree of crystallinity (*X*_c_) of the mixture can be calculated using eq 1:

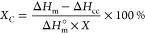
1

where Δ*H*_m_ is the melting enthalpy, Δ*H*_cc_ is the cold crystallization enthalpy, and = 93.6 J/g is the melting enthalpy of 100% crystalline PLA, and *X* is the fraction of PLA content.^34^

**Figure 2:**
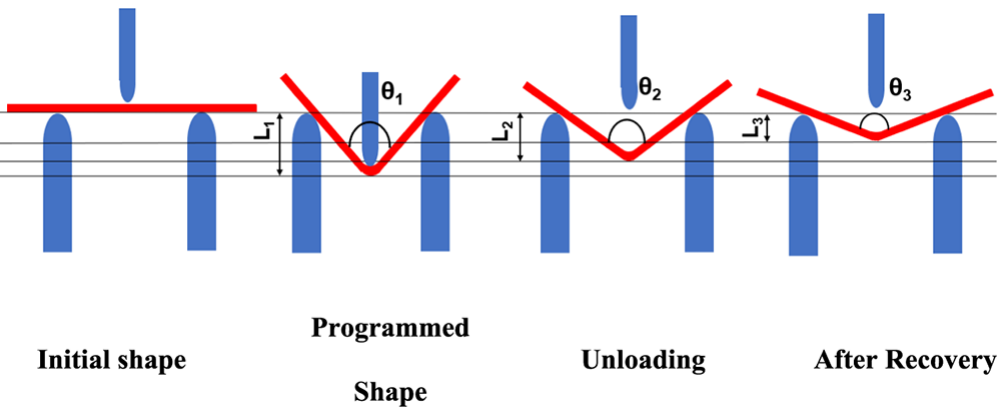
Illustration of shape memory test using three-point bending test.

- The tensile and three-point bending tests were conducted using an Instron universal testing system to investigate the mechanical properties of the prepared mixtures. Each test was repeated at least three times, and the results (i.e., tensile modulus, tensile strength, flexural modulus, and flexural strength) are reported as average values with standard deviations. Both tests were performed using a displacement control procedure at a displacement rate of 2 mm/min.
- Dynamic mechanical properties of the samples were examined in a single cantilever mode utilizing a dynamic mechanical analyzer METTLER-Toledo DMA/SDTA861. The samples were trimmed to dimensions of 10 × 3 × 0.5 mm^3^, and the properties were measured at a temperature range of 25–100 °C with a heating rate of 3 °C/min and a frequency of 1 Hz. DMA measurements provide valuable information on the *T*_g_ of SMP composites when it cannot be easily identified from DSC data, as the exact *T*_g_ value is overlapped by the wide range of thermal interaction.^3^

- As mentioned, SMPs can recover their original shape after being deformed by an external stimulus. The shape recovery properties of SMPs are different from the elastic recovery in hyper-elastic materials like rubbers. Polymers should memorize the original shape and should be able to keep the temporary shape to be categorized as SMPs. A quantitative measurement of materials’ shape memory properties, shape recovery ratio (*R*_r_), and shape fixity ratio (*R*_f_) can be used. For an ideal SMP, *R*_r_ = *R*_f_ = 1 means that SMP keeps the temporary shape and will recover the original shape at the end of the recovery process. However, in real applications, SMPs’ behavior differs from ideal cases; these ratios are less than 1. SMPs with higher values of these ratios show better shape memory properties. It is possible to combine those ratios and define a new parameter, i.e., shape memory index (*R*_i_) as *R*_i_ = *R*_r_ × *R*_f_. This study aims to find the optimum SMP mixture with CP capability at room temperature. Thus, the programming process of the shape memory test will be done at room temperature. Three-point bending, as a simple and basic way, is employed for the programming of the samples. The samples used for bending tests are used for the shape recovery test. As shown in Figure 2, the middle of samples is deformed downward (L_1_) at room temperature, which inclined the ample by the angle θ_1_, i.e., programming angle. Then the load is removed, and the sample recovers some part of the applied deformation elastically, thus, the midpoint deflection reduces to L_2_, and the angle decreases to θ_2_. The deformed sample is then heated in a water bath at a temperature higher than the polymer’s *T*_g_, approximately 80 °C, for 1 min. It will recover some part of the deformation, and the midpoint deflection reaches L_3_, and the final angle will be θ_3_. The shape fixity ratio and shape recovery ratio can be calculated as

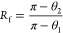
2
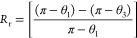
3

### 2.4 Finding the Optimum Mixture Using Gray Relational Analysis (GRA) Method

After the characterization of the prepared mixtures, the next step is to find the mixture with the optimum properties. The desired mixture should have high mechanical properties and flexibility at room temperature and high shape recovery properties. It is worth mentioning that increasing the TPU content in the mixture increases the flexibility and CP capability of the mixture; however, it decreases the mechanical strength of the mixture. Thus, optimization is a multiobjective process, and there should be a trade-off between the outputs to find the optimum mixture. The input is the TPU content of the mixture, and the outputs are the tensile modulus, tensile strength, shape fixity, and shape recovery ratio. The gray relational analysis (GRA) is generally used to combine all the considered outputs into a single value that can be used as the single characteristic in optimization problems. This way, the multiobjective optimization problem reduces to a single output optimization problem. In the first step, the signal-to-noise (S/N) ratio is calculated for each of the outputs. The S/N ratio can be defined in different ways as:^35^
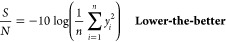
4
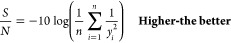
5in which *y*_*i*_ is the *i*th experiment at the test and *n* is the total number of trials in the tests. Since the data may have different scales, the next step in using GRA is data preprocessing and normalization. Based on the desired goal, each output should be normalized in the range of 0–1, where 1 is the best case and 0 is the worst case. If the target value of the original sequence has the characteristic of “lower-the-better”, the normalization should be as:^30^
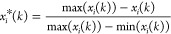
6and if the target value of the original sequence has the characteristic of “higher-the-better”, the normalization should be as:^30^
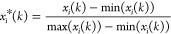
7where  and *x*_*i*_(*k*) are the *i*th normalized and original output, respectively. In this work package, there are four outputs (i.e., tensile modulus, tensile strength, shape fixity, and shape recovery ratio), and for each output, there are eight samples; thus, *i* = 1, 2, 3, 4 and *k* = 1, 2, ··· , 8. A gray relational coefficient (GRC) can be calculated as:
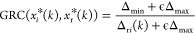
8where  is reference sequence, and Δ_r*i*_(*k*) represents the deviation sequence of  and , ϵ = 0.5 is the distinguishing coefficient, and the other parameters are defined as:^36^
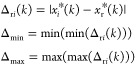
9

**Figure 3:**
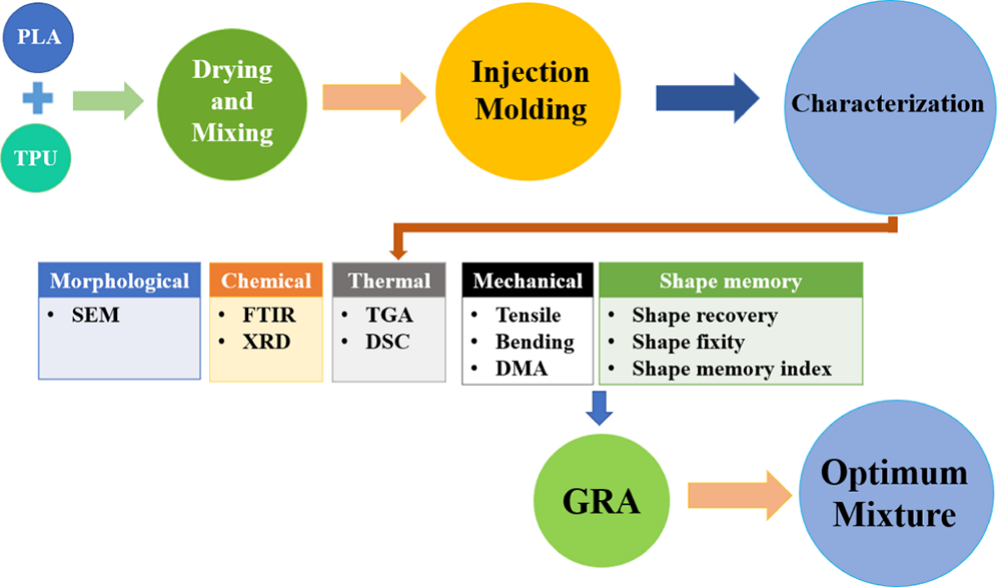
Outline of this study.

The reference sequence is considered to be the sequence with the maximum normalized value, i.e., . Then the average gray relational grade (GRG) can be obtained as:
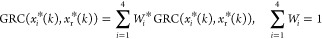
10where *W*_*i*_ is the corresponding weight of the *i*th output. The value of the weights should be defined based on the optimization goal. For instance, equal weight should be used if all of the inputs have the same priority. However, one or two of the outputs are more important. Their weight should be higher than that of the other. Then the considered samples are ranked based on the GRG values, and the optimum parameters are considered to correspond to the trial with the maximum GRG value. The general procedure of this research is shown in Figure 3.

## 3 Results and Discussion

**Figure 4:**
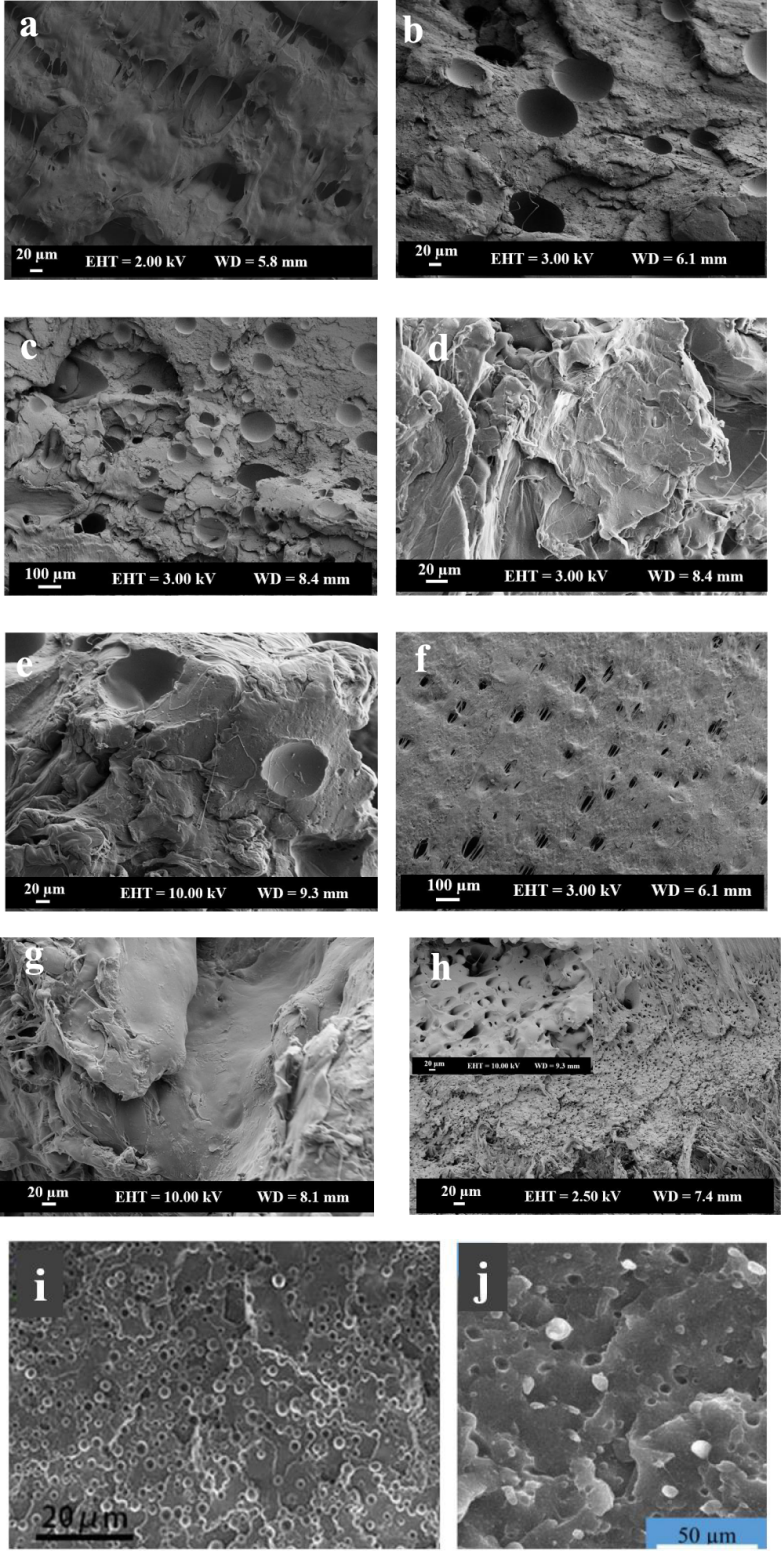
SEM images of (a) PLA, (b) PLA/TPU 5, (c) PLA/TPU10, (d) PLA/TPU15, (e) PLA/TPU20, (f) PLA/TPU30, (g) PLA/TPU40, (h) PLA/TPU50, and (i) PLA80/TPU20,^26^ adapted with permission under a Creative Commons Attribution 4.0 International License, all rights reserved. (j) PLA50/TPU50,^28^ adapted with permission. Copyright 2024, Elsevier B.V., all rights reserved.

SEM analysis was carried out to study the distribution of the PLA and TPU polymer blend. In Figure 4a–h, SEM images depict pure PLA and PLA/TPU blends containing varying TPU concentrations (5, 10, 15, 20, 40, and 50 wt %). A homogeneous structure of PLA/TPU is evident across all compositions, with no observable phase separation between PLA and TPU. The SEM analysis reveals a uniform distribution of TPU within the PLA matrix for all SMPCs. In recent studies of PLA/TPU blends, significant phase separation between PLA and TPU is evident in SEM images. Consequently, researchers concluded that TPU and PLA are immiscible. To address this, various works have recommended the addition of a compatibilizer to enhance the homogeneity of the PLA/TPU mixture.^4,14,24,3,37−41^ In the SEM images of PLA/TPU mixtures presented herein, no discernible phase separation is evident, contrasting with the SEM results reported in the literature. This comparison is illustrated in Figure 4i,j, where findings (Figure 4a–h) differ from those reported by Obaidur Rahman et al.^26^ (PLA80/TPU20) and Rahmatabadi et al.^28^ (PLA50/TPU50). Unlike their observations, this study image does not reveal any distinct phase separation between the PLA and TPU components indicating the successful mixing of the two components and enhancement of the mixture compatibility. Most probably, one of the main reasons for this is using a high shear thermo-kinetic mixer, while in the mentioned works the twin-screw extrusion method has been used for mixing the polymers. Ariturk et al.^42^ also noted that a high mixing speed in the thermo-kinetic mixer induces shear, which results in the homogeneous processing of even incompatible materials without the requirement for external compatibilizers. As a result of a homogeneous mixture of PLA and TPU or any other SMPCs which shows the enhanced compatibilization of blends, composites strength increases and thermal properties enhance which can result in SMPs with higher shape recovery ratio, young modulus, and ultimate stress and toughness.^2,24,27,43^ For example, based on the mechanical analysis results, a lower decrease in mechanical properties was achieved with the addition of TPU to PLA compared to the results reported in the literature. This discrepancy is thoroughly discussed in the mechanical results discussion in the results section. While, the DMA and DSC results reveal a slight reduction in the *T*_g_ of the SMCs compared to pure PLA, providing evidence for compatibility of blends,^12,44^ and XRD results are also in good agreement with other findings of this study.

**Figure 5:**
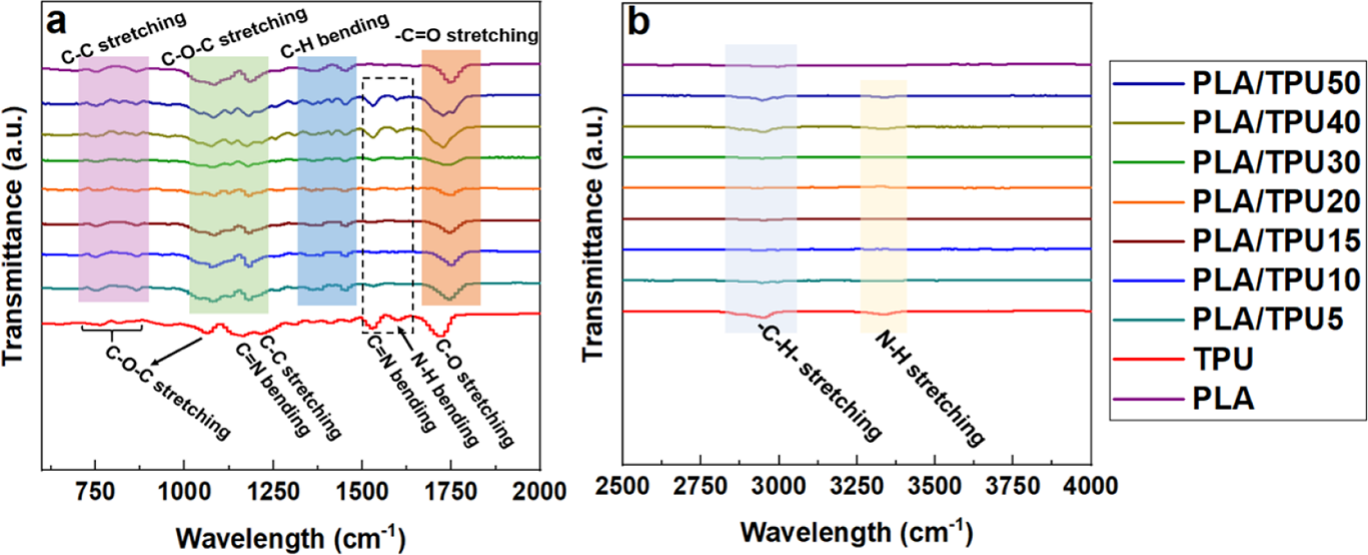
FTIR results of PLA/TPU mixture: (a) 600–2000 cm^–1^ and (b) 2500–4000 cm^–1^ wavenumbers.

Results of the FTIR analysis of PLA/TPU mixtures are presented in Figure 5. PLA has a characteristic C=O stretching bond at 1752 and 1654 cm^–1^ wavelengths as the most active site in PLA.^45,46^ C–H weak peaks around 3000 cm^–1^ wavelengths and peaks at 1452, 1383, and 1361 cm^–1^ belong to the stretching and bending of C–H bonds in PLA. Peaks at 1180–1040 cm^–1^ arise from −C–O–C- stretching vibrations.^47−49,50^ The absorption band at 3338 cm^–1^ in the spectrum of pure TPU corresponds to N–H stretching. Then the peaks at 2926 and 2843 cm^–1^ are related to the asymmetrical and symmetrical stretching in CH, CH_2_, and CH_3_ groups. The band around 1705 cm^–1^ is due to C=O stretching of TPU.^51^ At 1595 cm^–1^ is NH bending and aromatic C–C stretching, following 1529.6 cm^–1^ for C=N stretching and N–H bending, 1418 cm^–1^ for C–C stretching of the aromatic ring, 1309 cm^–1^ for C=N stretching, NH, 1220 cm^–1^ for C=N stretching, NH, and 1164 and 1063 cm^–1^ for C–O–C stretching. The difference between the ester and the ether groups is in the frequency of the C–O–C bond stretching, which corresponds to the wavelength of 1100 cm^–1^ for the asymmetric ether and urethane stretching, or 1250 and 1150 cm^–1^ for the ester groups.^52^ FTIR results of PLA/TPU5, PLA/TPU10, PLA/TPU15, PLA/TPU20, and PLA/TPU30 blends show characteristic peaks mainly of PLA functional group wavelength except having a peak at 1596 cm^–1^ arising from bending of NH bonds of TPU. Also, the intensity of peaks for composite polymers has changed compared to pure PLA and TPU. As the ratio of TPU has increased in PLA/TPU40 and PLA/TPU50, a shift can be seen toward characteristic peaks of the TPU FTIR spectrum. These results suggest the successful melt blending of PLA/TPU mixtures and existence of physical interaction between PLA and TPU blends since there is no evidence of chemical reactions during the blending process and no newly identifiable chemical bonds.^24^

**Figure 6:**
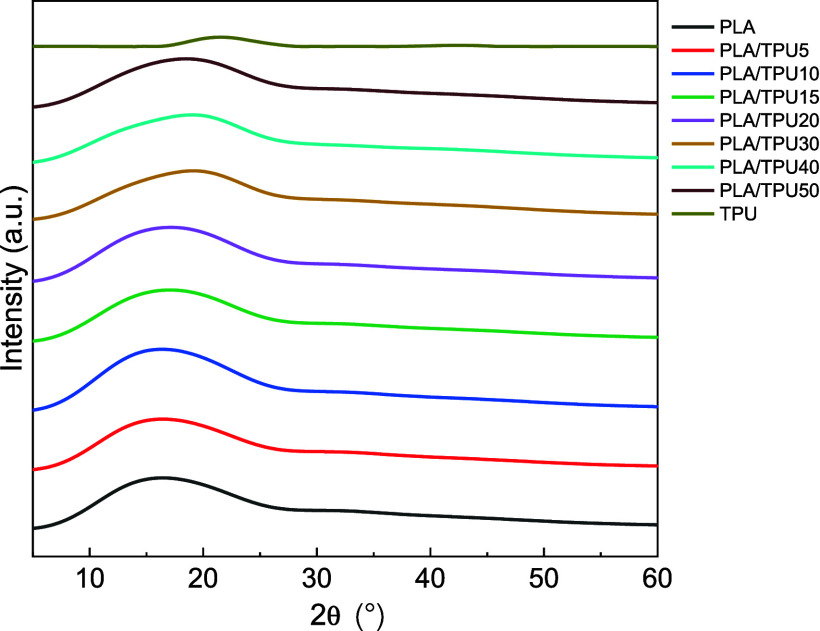
XRD results of PLA, TPU, and PLA/TPU blends.

Figure 6 shows the results of the XRD analysis of the mixtures with different amounts of TPU. Results prove that the PLA, TPU, and PLA/TPU blends are highly amorphous and have low crystallinity.^53,54^ PLA showed a broad peak around 2θ = 16.5° assigned to the crystalline phase α. TPU showed a broad peak at 2θ = 21°, which is relevant to the existence of a short-range regularly ordered structure of both hard and soft domains along with a disordered structure of the amorphous phase of the TPU matrix.^55,56^ In blends with higher TPU ratio (PLA/TPU30, PLA/TPU40, PLA/TPU50), it seems the PLA peak at 16.5° shifted to TPU peak at 21° while PLA/TPU blends with 5,10,15, and 20 wt % TPU showed 2θ peak at 16.5°. The XRD results indicated that regardless of the TPU content, the PLA/TPU blends primarily exhibit the same characteristic peaks as pure PLA. It suggest that TPU did not affect the basic structure of PLA.^26^ It may also be because of the lower ratio and weaker signal associated with the TPU content or homogeneous distribution of TPU in the PLA matrix.^57^

**Figure 7:**
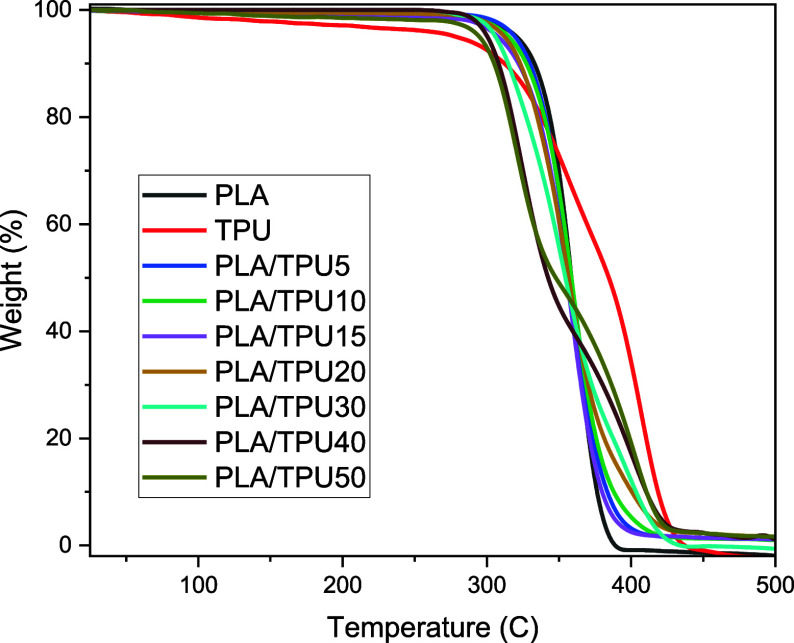
TGA results of PLA, TPU, and PLA/TPU blends.

TGA was carried out to measure and determine the degradation temperatures of PLA, TPU, and their blends in different ratios. Figure 7 and Table 1 give the TGA results and the initial and final degradation temperatures. As shown in Table 1, PLA’s initial degradation starts at 330 °C, and the complete degradation is at 376 °C. The initial degradation temperature of TPU starts at 306 °C with a final degradation temperature of about 421°. In PLA/TPU blends with 5,10, 15, 20, and 30 wt % TPU, initial and final degradation temperatures are between PLA and TPU with a trend that as the ratio of TPU in blends increases, the initial degradation temperature of blends decreases. The decomposition temperature of PLA/TPU40 and PLA/TPU50 is lower than that of TPU, and as the ratio of TPU has increased in PLA/TPU40 and PLA/TPU50, two-stage degradation can be observed due to the high ratio of TPU. PLA/TPU blends’ final degradation temperature of PLA/TPU blends is 6–12 °C higher than pure PLA. SMP composite’s lower degradation temperature than each polymer content has been reported in related references.^3,58^

**Table 1:** Initial and Final Degradation Temperatures Were Derived from TGA Results

sample	*T*_i_ (°C)	*T*_f_ (°C)
PLA	330	376
TPU	306	421
PLA/TPU5	327	382
PLA/TPU10	321	388
PLA/TPU15	317	381
PLA/TPU20	316	387
PLA/TPU30	308	384
PLA/TPU40	299	384
PLA/TPU50	298	375

**Figure 8:**
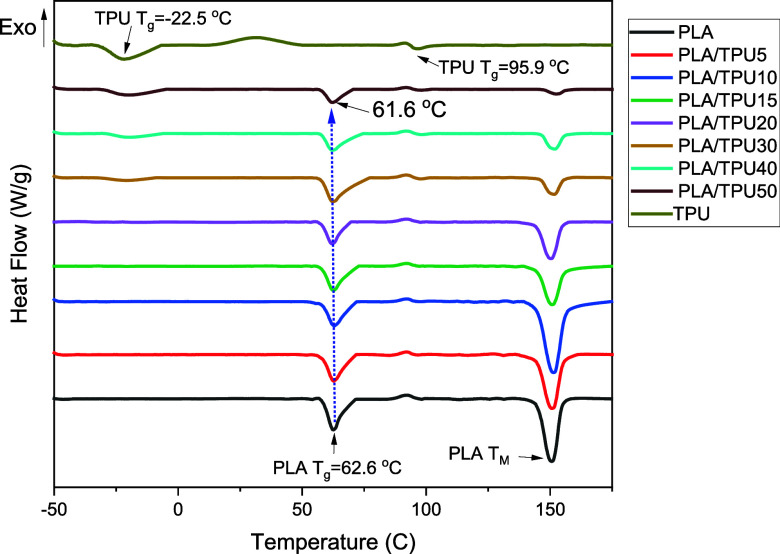
DSC results of the second heating cycle for PLA, TPU, and PLA/TPU mixture.

**Figure 9:**
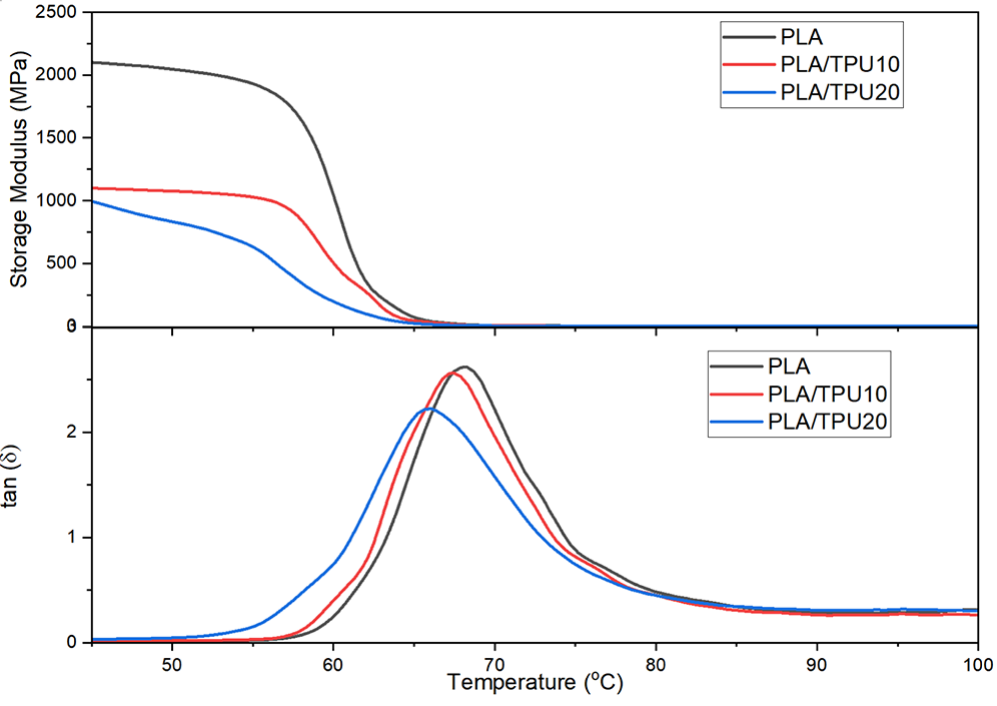
DMA results for PLA, PLA/TPU10, and PLA/TPU20.

Figure 8 presents the DSC results of PLA, TPU, and PLA/TPU blends. Pure PLA shows a typical semicrystalline curve with low crystallinity, and TPU shows a plain curve. PLA *T*_g_ is 62.6 °C, and results proved that increasing the amount of TPU in the mixture does not affect the *T*_g_ of the mixture significantly; the mixture *T*_g_ decreased to 61.6 °C for PLA/TPU50. This small shift in *T*_g_ suggests the compatibility of PLA/TPU blends.^12^ Furthermore, in the DMA results, peaks in tan δ are linked to the *T*_g_, providing greater accuracy compared to DSC data, which can be susceptible to overlap in a wide range of thermal interactions.^3^ Based on the DMA findings (Figure 9), the introduction of 10 and 20% TPU resulted in a slight decrease in the *T*_g_ of pure PLA from 68 to 67.5 °C and 65.5 °C, respectively.^12,34^ Additionally, the presence of a singular peak in tan δ serves as further evidence of compatibility between the PLA and TPU content.^59^ Moreover, TPU shows a *T*_g_ arising from its soft segment at −23 °C and another from its hard segment at 96 °C. In the mixture with a low amount of TPU, these *T*_g_ peaks are not visible; however, when the TPU amount increases, i.e., 30, 40, and 50 wt %, these peaks are visible.^37^ TPU during the second heating cycle depicts both melting and subsequent crystallization peaks between 0 and 50 °C, instead of the expected melting peak of the soft segment,^60,61^ suggesting a complex thermal behavior. This may be due to recrystallization of the soft segments during heating. As the TPU is heated, the soft segments may first melt and then quickly recrystallize due to sufficient segmental mobility, forming more ordered crystalline structures before fully melting. This behavior can also result from phase separation and reorganization of the soft and hard segments, influenced by the specific chemical composition and molecular weight distribution of the TPU. Additionally, the thermal history and cooling rate of the sample might have led to the formation of stable crystalline structures, which melt and recrystallize within the observed temperature range.^62−64^ Pure PLA and PLA/TPU mixtures show weak cold crystallization peaks around 92 ± 1 °C, a process associated with the exothermal self-nucleation of the crystalline phases above the glass transition temperature. Also, the melting temperature (*T*_m_) of PLA at 150.64 °C did not vary much across all TPU concentrations. However, the cold crystallization enthalpy exhibited a decrease with the addition of TPU, arise from its rubbery segment.^12^ Furthermore, the melting enthalpy and crystallization ratio of the PLA/TPU blends decreased with the addition of 5, 15, 20, 30, 40, and 50% TPU to pure PLA. However, the introduction of 10% TPU shows an increase in the melting enthalpy and crystallization ratio. Summary of *T*_g_, *T*_cc_, *T*_m_, cold crystallization enthalpy, melting enthalpy, and crystallization ratio of pure PLA, TPU, and their blending are given in Table 2.

**Table 2:** Glass Transition (*T*_g_), Cold Crystallization (*T*_cc_), Melting Point (*T*_m_) Temperatures, Cold Crystallization, Melting Enthalpy, and Crystallization Ratio of PLA, TPU, and Their Blends Were Derived from DSC Results

samples	*T*_g_ (°C)	*T*_cc_ (°C)	*T*_m_ (°C)	cold crystallization enthalpy (J/g)	melting enthalpy (J/g)	crystallization ratio (%)
PLA	62.6	92.47	150.64	0.2887	5.30	5.38
TPU	–22.5095.91					
PLA/TPU5	62.70	93.11	150.55	0.1107	4.56	5.04
PLA/TPU10	62.75	92.69	151.01	0.1477	6.15	8.37
PLA/TPU15	62.55	93.2	149.25	0.1460	2.44	2.9
PLA/TPU20	62.05	92.41	151.49	0.2137	1.69	1.98
PLA/TPU30	–23.0562.01	92	151.92	0.2124	1.49	1.97
PLA/TPU40	–23.5761.56	91.78	152.24	0.21	1.32	1.99
PLA/TPU50	–27.8561.61	92.35	153.08	0.1614	0.423	0.56

Figure 9 represents the thermomechanical behavior of the PLA, PLA/TPU10, and PLA/TPU20 mixtures using the DMA test. Storage modulus and loss tangent, tan(δ) which is the ratio of loss modulus and storage modulus, have been reported in this figure. Results show that PLA, PLA/TPU10, and PLA/TPU20 mixtures have *T*_g_ of 68.1, 67.30, and 65.85 °C, respectively. The slight shift of SMP’s *T*_g_ to a lower temperature than PLA suggests the compatibility of PLA/TPU blends.^44^ The *T*_g_ values obtained from the DMA test are different from those extracted from the DSC test resulting from the frequency factor of the analysis techniques.^65^

Moreover, the height of the tan(δ) peak decreases as the TPU content of the mixture increases. This reduction in the peak height is a sign of improvement in the polymer phase interactions. It can also show that damping-ability of the SMP mixtures decreased owing to the PLA–TPU interactions.

All of the SMPs have high storage modulus values below their *T*_g_; however, above their *T*_g_, the storage modulus decreased significantly, as expected. As expected from mechanical test results, PLA has the highest storage modulus. Increasing the TPU content decreases the mixture’s storage modulus due to the lower intrinsic modulus of TPU in the rubbery region at a temperature above its *T*_g_ of −22 °C.

**Figure 10:**
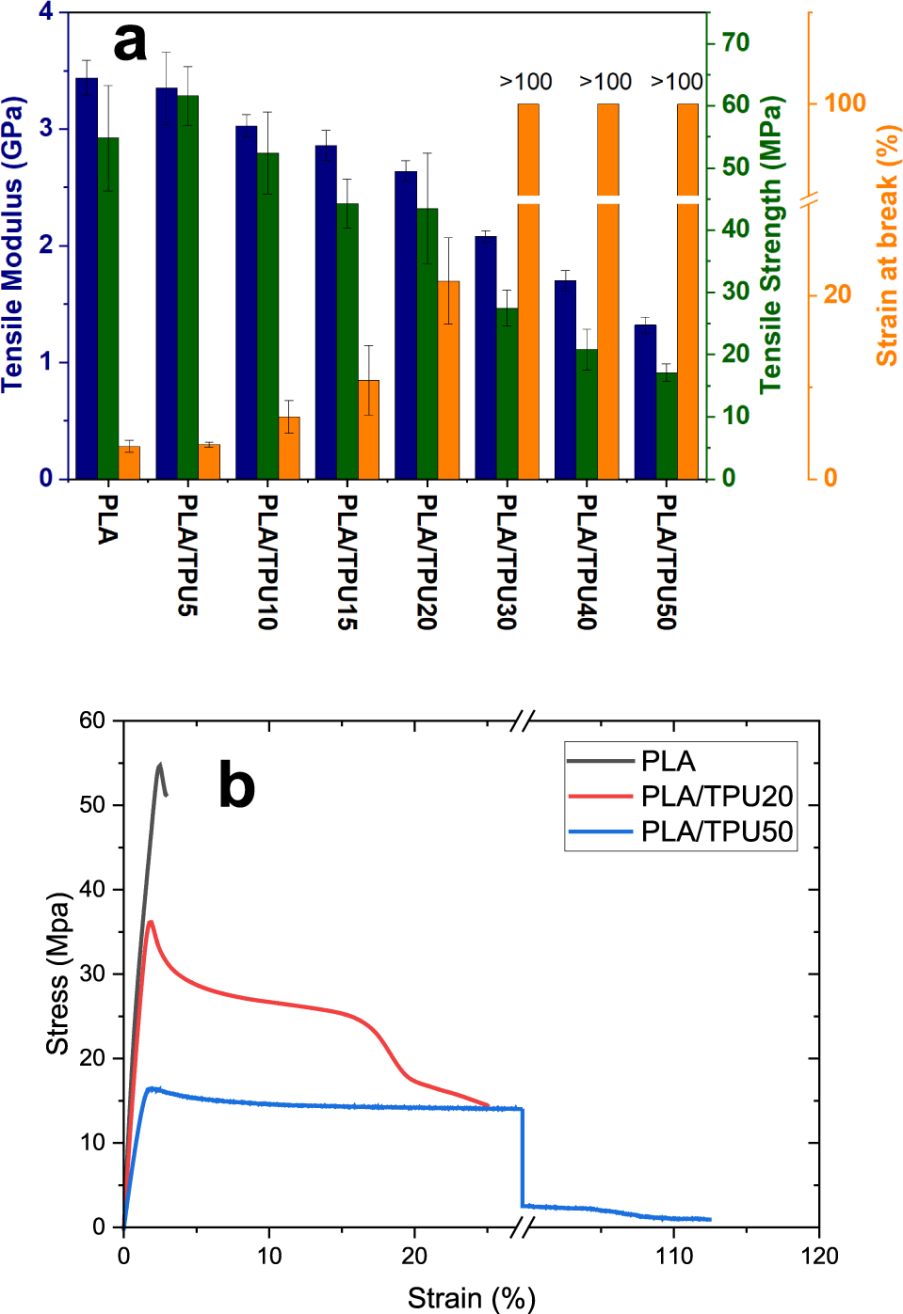
(a) Results of tensile tests of SMP mixture and (b) stress–strain curve of PLA, PLA/TPU20, and PLA/TPU50.

Figure 10 shows the results of the tensile tests of the SMP mixtures. In Figure 10a, the elastic modulus, tensile strength, and strain at the break of the mixture with different contents of TPU are presented. Pure PLA has modulus, strength, and strain break of 3.44 GPa, 54.8 MPa, and 3.55%, respectively, which is in good agreement with the reported result by the other researchers. As expected, increasing the TPU content in the mixture decreases the elastic modulus and strength however increases the strain at the break of the mixture. For instance, adding 20% of TPU to PLA leads to modulus, strength, and strain at breaks of 2.64 GPa, 43.5 MPa, and 21.6%, respectively. As can be seen, the modulus and strength decreased 25 and 21%, respectively; however, the strain at break increased by 500%. For the mixture with higher content of TPU, i.e., PLA/TPU 30, 40, and 50, the strain at break is higher than 100%, which shows that the material is completely flexible at room temperature. However, the mechanical properties reduce dramatically; for instance, the PLA/TPU 30 has a modulus and strength of 2.01 GPA, 27.5 MPa, which shows 42 and 50% reduction compared to pure PLA. The PLA/TPU50 mechanical properties, i.e., modulus and strength, have been reduced by 61 and 69%, respectively. Upon comparison of the reduction in tensile strength resulting from the addition of TPU content in our prepared SMPCs with relevant studies employing the same PLA/TPU ratio, it becomes apparent that the decrease ratio in our samples is lower. This likely arises from the successful blending method and compatibility between PLA and TPU content. In the study conducted by Kahraman et al.,^66^ the introduction of 5 and 15% TPU (ester based, Ravathane 165 A85) to PLA (Ingeo 4060D) resulted in a reduction of tensile strength by approximately 20 and 36%, respectively. In the studies conducted by Jing et al.,^22^ the incorporation of 20% TPU led to a reduction in the tensile strength of PLA by approximately 30.7%. In another study conducted by Jafari Horastani et al.,^27^ the addition of 20 and 40% TPU (EPAMOLD 665A26-T3165EX) resulted in a reduction of 33 and 68.8% in the tensile strength of PLA, respectively. Summary of the discussed results is given in Table 3

**Table 3:** Summary of the Tensile Strength Results from This Study and Relevant Literature

	present work		Kahraman et al.^66^		Jing et al.^22^		Jafari Horastani et al.^27^	
mixture	tensile strength (MPa)	change (%)	tensile strength (MPa)	change (%)	tensile strength (MPa)	change (%)	tensile strength (MPa)	change (%)
pure PLA	54.8		56		65		62	
PLA/TPU5	62	+13	44	–21				
PLA/TPU15	45	–17	36	–35.7				
PLA/TPU20	43.5	–21			45	–30.7	42	–32.2
PLA/TPU40	20.5	–62					19.33	–68.8

Tensile test results indicate that mixtures containing 5, 10, 15, and 20% TPU content have comparable mechanical properties with respect to pure PLA. However, the mixture with 5 and 10% TPU shows brittle behavior at room temperature, which is unsuitable for CP. Thus, regarding the tensile test results, PLA/TPU15 and PLA/TPU20 are possible candidates for the mixture with the optimum properties. Considering the shape memory test results, the best mixture will be selected.

**Figure 11:**
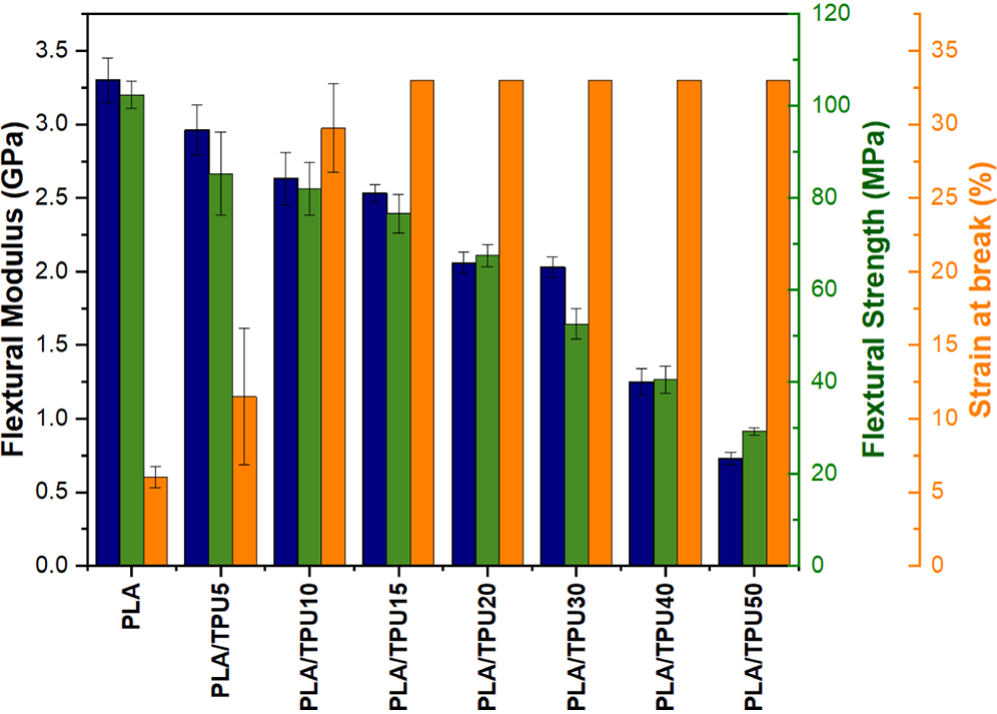
Results of flexural three-point bending test.

Figure 11 shows the three-point bending test results. Again, adding more TPU to the mixture increases its flexibility; however, its mechanical properties decrease. The PLA and PLA/TPU5 show a completely brittle behavior at room temperature, as observed in the tensile test. However, the mixture with a TPU content of more than 15% tolerates higher strains at room temperature. The final flexural strain for the mixture with TPU content of 20, 30, 40, and 50 is 32% because, after this strain, the samples started to slide and the test was stopped. There is a correlation between the results of the tensile test and the flexural test; thus, for finding the optimum mixture, only the tensile properties are considered.

**Figure 12:**
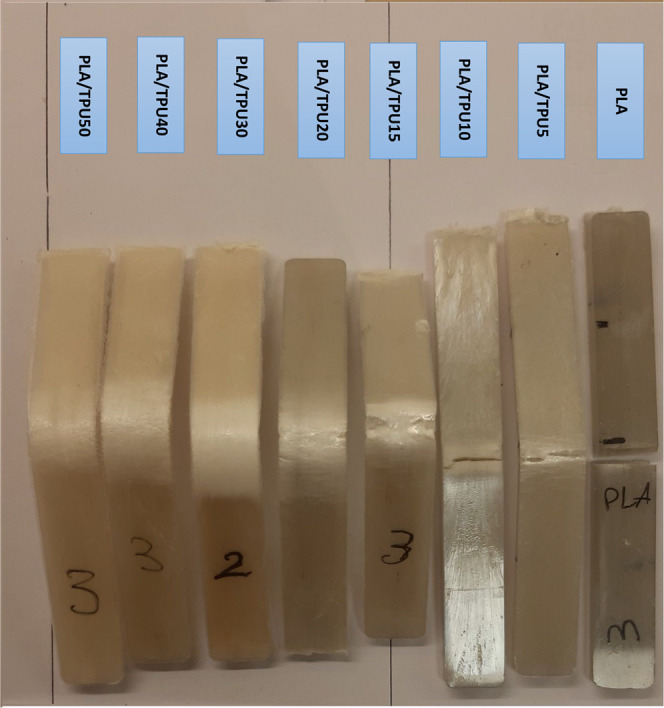
Deformed samples at the end of the flexural test.

The deformation at room temperature during the flexural test is the programming step in the shape recovery test. The main goal of this study is to prepare an SMP mixture with a CP capability at room temperature. The PLA and PLA/TPU5 break at low strain; thus, they are unsuitable for CP. The PLA/TPU10 breaks at a strain of 30%, and the final strain of the rest of the mixtures is 32% (due to sample slippage, further deformation was not possible). This high strain value proves their flexibility at low temperatures; however, the PLA/TPU10 and PLA/TPU15 samples cracked at this strain. This means that even if they recover their shape, they cannot be used again due to this crack. Figure 12 shows the deformed samples at the end of the flexural test. The cracks are visible, and as seen, PLA/TPU20, 30, 40 and 50 do not have any cracks, and the outer surface of the samples does not have any cracks or imperfections. It again suggests that PLA/TPU20 is a possible candidate for the optimum mixture.

### 3.1 Shape Memory Test Results

**Figure 13:**
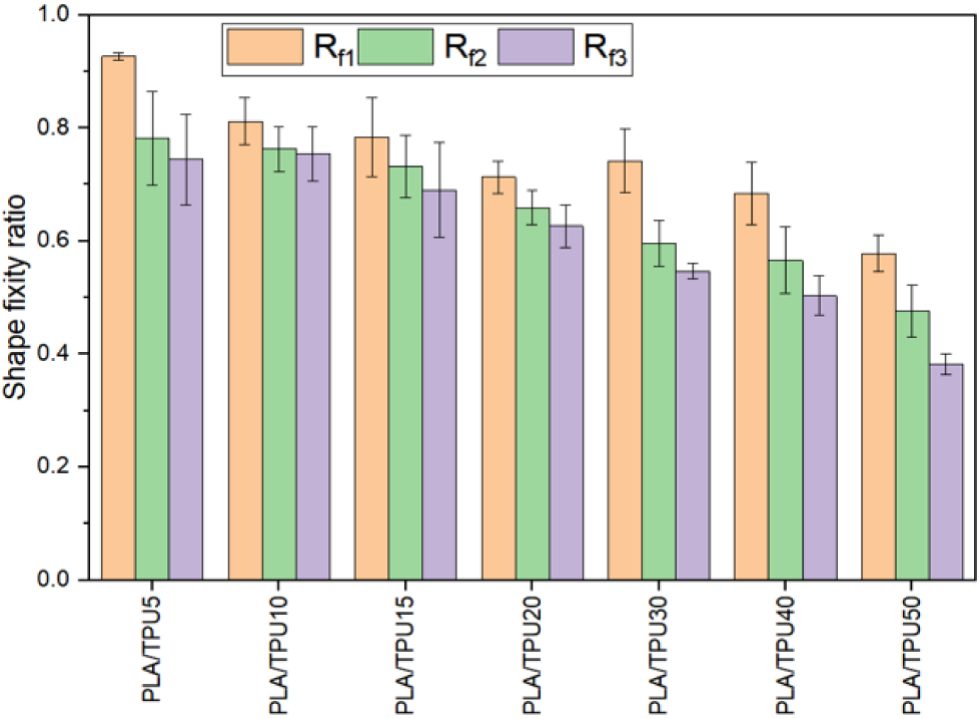
Shape fixity ratio of the samples at different time.

Shape memory test consists of programming and recovery steps. In this research, only CP is considered; thus, the deformation applied to the samples during the flexural test can be considered a programming step. In the PLA/TPU blend via CP, the SME is linked to the energy dissipation process in the rubber-toughened structure. Elastic property differences between TPU and PLA play a pivotal role in this mechanism. When subjected to an external force at room temperature, the stress concentration first initiates in the proximity of TPU domains due to higher elasticity. As the stress concentration reaches a critical level, debonding occurs at the interface between the TPU and PLA, leading to void formation. This debonding process releases local triaxial stress, dissipating energy. Concurrently, the PLA amorphous domain between TPU particles undergoes deformation alongside the debonding process. The PLA utilized in this study is a semicrystalline polymer, and its crystalline domain exhibits limited mobility at room temperature, serving as pivotal switch points for regulating shape fixity under external forces. Conversely, the amorphous phase, deforming with TPU, functions as nodal points determining the permanent shape. Upon removal of the external force, the temporary shape is retained and the elastic energy becomes stored within the system. When the temperature increased beyond the *T*_g_ of PLA, the molecules regained mobility, resulting in the recovery to their original shape and dissipating the stored energy.^12,22,24,67^ The shape fixity ratio was calculated using eq 2 by measuring the angle between the sides of the samples. It is worth mentioning that the polymers generally show time–temperature-dependent behavior. In other words, their response depends not only on the environment temperature but also on the time of loading or unloading. When the load is removed from the deformed samples, they show an immediate elastic recovery, after a while, when the deformed sample stays at room temperature, it recovers some part of deformation in an isothermal process, i.e., isothermal recovery. To investigate this phenomenon, the shape fixity of samples was measured at three different times, i.e., *R*_f1_ immediately after unloading, *R*_f2_ 1 h after unloading, and *R*_f3_ 12 hours after unloading, see Figure 13. As expected, as time passes, the shape fixity decreases due to isothermal recovery of the deformation; however, the rate of change decreases as time passes. For instance, for the PLA/TPU5, *R*_f1_ = 0.92, *R*_f2_ = 0.78, and *R*_f3_ = 0.74. it means that the fixity ratio decreased by 15% 1 h after unloading while it decreased by 6% after 12 hours. Results show that increasing the amount of TPU in the mixture decreases the shape fixity ratio; in other words, the sample cannot remember the programmed shape well. It is due to the elastic behaviors of TPU arising from its segmented structure, which acts as physical cross-links.^24,68^ A high shape fixity ratio is desired in applications such as packaging or deployable structures. However, as stated before, in structural applications, a lower shape fixity is preferred. Thus, increasing the TPU content in the mixture makes it more suitable for this application from a shape-fixity point of view. But other factors like the recovery and mechanical properties should also be considered.

**Figure 14:**
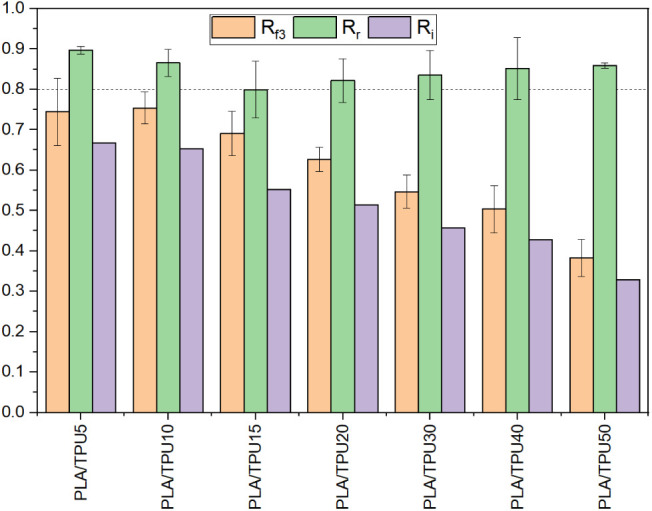
Shape recovery ratio and shape memory index of the samples.

**Figure 15:**
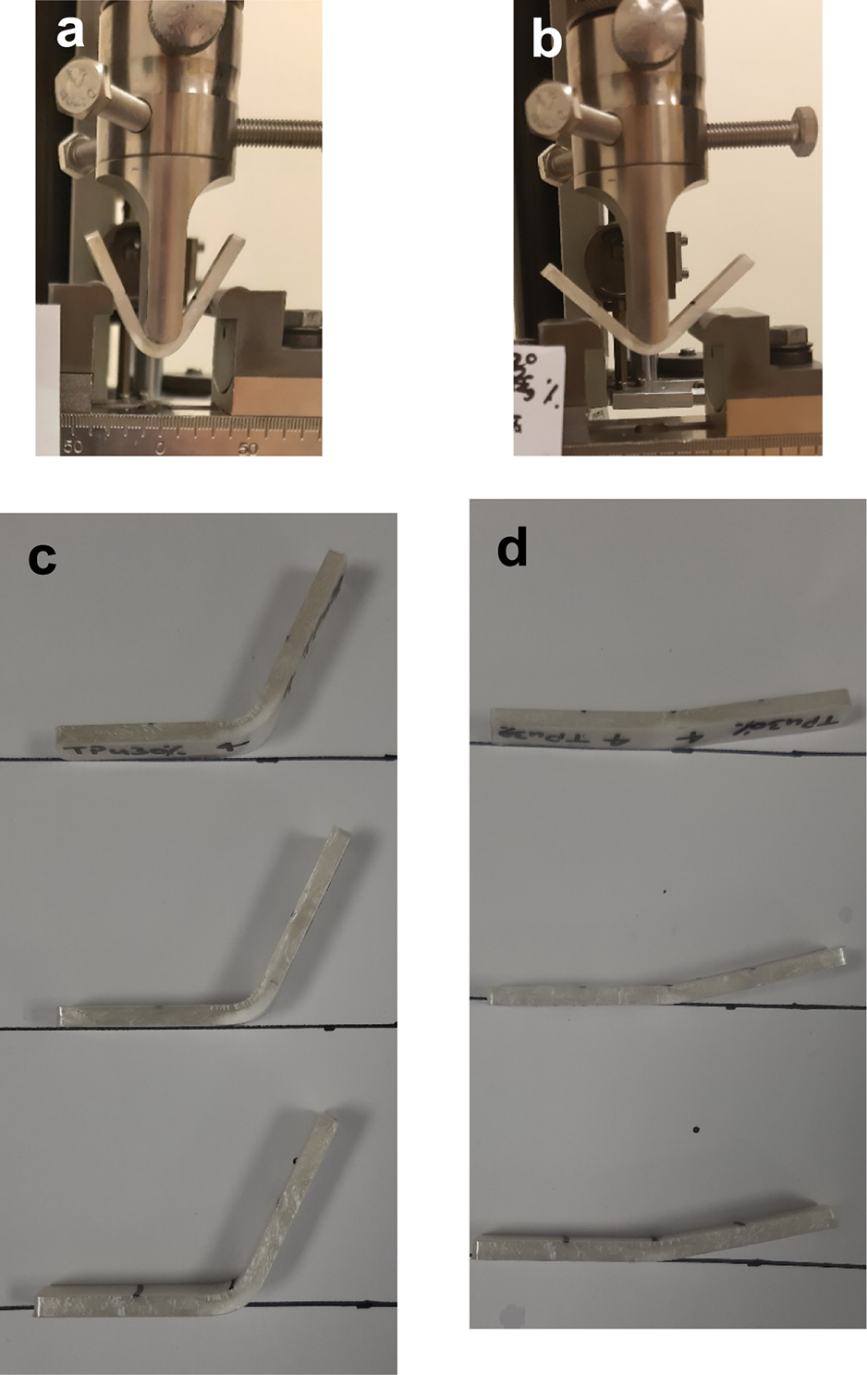
PLA/TPU30, shape memory test process, (a) programming at room temperature and end of loading process (ε *=* 32%), (b) end of unloading process, *R*_f1_ is calculated based on this angle, (c) samples’ shape 12 hours after programming (*R*_f3_), and (d) samples after shape recovery test.

In the next step, a shape recovery test of the samples was done. Regarding the DSC and DMA results, the recovery temperature was chosen to be 80 °C, higher than the mixtures’ *T*_g_. At the end of the recovery test, the final angle was measured, and using eq 3, the shape recovery ratio was calculated. Results of the recovery test, i.e., the shape fixity (*R*_f_), shape recovery ratio (*R*_r_), and shape memory index (*R*_i_) are presented in Figure 14. As an example, the programming and recovery test process for PLA/TPU30 samples is presented in Figure 15. Results show that all mixtures have a shape recovery ratio of more than 0.80, i.e., 80%. Results show that the addition of TPU does not change the shape recovery of the mixture that much; however, it significantly affects the shape fixity ratio and shape memory index. Similar results have been reported previously by other researchers.^23^ It is worth mentioning that although the shape fixity and shape recovery ratio are reported for PLA/TPU5 and PLA/TPU10, these samples are broken and cannot be used after the recovery process. Although PLA/TPU15 did not break at the end of loading, some cracks appeared in the deflection point at the end of loading, which means that it cannot be reused after recovery. Moreover, since PLA is brittle at room temperature, it did not show shape memory behavior at room temperature; thus, PLA is not included in the shape recovery test results.

Results show that by increasing the TPU content of the mixture, the shape recovery decreases first and then increases so that PLA/TPU15 has the minimum shape recovery ratio. It should be noted that in studies focused on warm or hot programming, the shape recovery ratio decreases as the TPU content increases.^12^ However, in cold programming, increasing the TPU content of the mixture leads to small increase in the shape recovery ratio, as reported by other researchers.^23^ One reason can be the high shape memory properties of pure TPU due to its segmented structure serving as physical cross-links to possess strong elastic behaviors. Thus, the recovery ratio increased at higher TPU content through a higher degree of physical cross-links. This was also attributed to TPU acting as a stress concentrator to avoid the premature failure of PLA at room temperature and good compatibility of PLA and TPU.^23,69^

**Figure 16:**
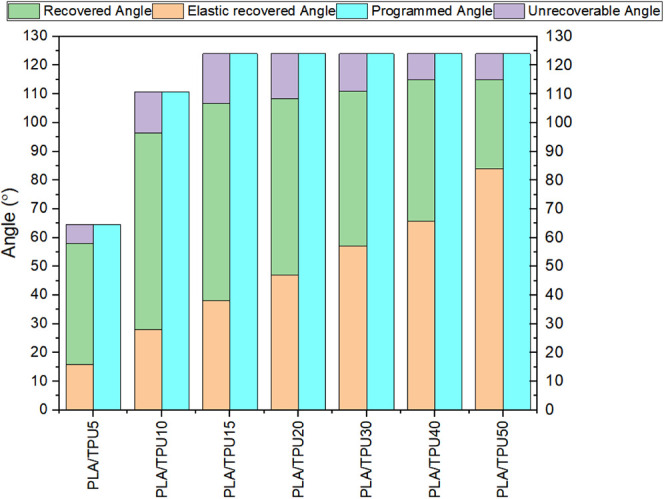
Details of the shape recovery test.

Although the shape fixity and shape recovery ratio give insight into the shape memory properties of the mixture, they cannot represent all aspects of SMP behavior. For instance, based on eq 3, the shape recovery ratio is defined based on the programming and final angles. In our case, the programming angle is equal for all mixtures, and the final angle is almost the same for all mixtures. That is why the shape recovery ratio does not vary much with changing the TPU content of the mixture. Figure 16 shows more detail about each mixture’s whole programming and recovery process. This figure presents the programmed angle, elastically recovered angle, recovered angle, and unrecoverable angle. Although the results of Figure 14 showed that increasing the TPU content of the mixture does not lead to a significant change in the mixture shape recovery ratio, it increases the elastically recovered angle and decreases in recovered angle. In other words, although the mixtures have the same shape recovery ratio, their ability to recover deformation decreases as the TPU content of the mixture increases.

### 3.2 Finding the Optimum Mixture Using Gray Relational Analysis

In this section, the optimum mixture among all of the considered ones is chosen with the help of GRA. The importance of optimization lies in achieving a PLA/TPU blend that meets the specific requirements of its intended application. While it is true that actual applications play a pivotal role in dictating material requirements, optimization serves as a systematic approach to fine-tune the blend to its best possible performance across various parameters. The application of GRA in this study facilitates identification of the optimal TPU content for the mixture. This optimization process is essential, as it enables a better understanding of the relation between mechanical and shape memory properties, providing insights that contribute to the tailored development of the blend to meet the specific demands of its intended application. Herein, the input parameter is the TPU content of the mixture, and the outputs are mechanical properties (i.e., tensile modulus (*E*), tensile strength (σ_u_), and strain at break (ε_b_)) and shape memory properties (i.e., shape recovery and shape fixity ratio). As mentioned before, since there is a correlation between the tensile and flexural test results, only the tensile test is considered in the optimization process. The raw output data are presented in Table 4. It is worth mentioning that the optimization goal for mechanical properties and shape recovery ratio is higher-the-better, while the shape fixity is the lowest-the-better. Because in the load-bearing applications, despite packaging application, it is desired to have the lowest possible shape fixity and highest possible recovery ratio. Thus, the S/N ratios for mechanical properties and shape recovery ratio are calculated using eq 5, and the S/N ratio for shape fixity is calculated using eq 4. The normalized output and deviation sequences are presented in Table 5. The GRC and GRG are calculated using eqs 8 and 10 and presented in Table 6. The GRC values in Table 6 are modified to consider the design constraints for practical applications. For instance, the mechanical properties of the desired mixture should not be less than half of the pure PLA; thus, the GRC values of a mixture with tensile strength less than 30 MPa are set to zero. The same happened for mixtures with a modulus less than 1.75 GPa and strained at a break less than 10%. For the shape recovery, since the PLA/TPU5, PLA/TPU10, and PLA/TPU15 samples break or crack during the programming process, their shape recovery’s GRC is set to zero. Based on the GRG values, the PLA/TPU20 has the highest GRG. The PLA/TPU20 properties are *E* = 2.64 GPa, σ_u_ = 43.50 MPa, ε_b_ = 21.60%, *R*_f3_ = 0.62, and *R*_r_ = 0.82.

**Table 4:** Raw Output and Their S/N Ratio

	raw output					S/N ratio				
sample name	*E* (GPa)	σ_u_ (MPa)	ε_b_ (%)	*R*_f3_	*R*_r_	*E*	*σ*_u_	*ε*_b_	*R*_f3_	*R*_r_
PLA/TPU5	3.35	61.60	3.77	0.74	0.89	10.50	35.79	11.53	2.62	–1.01
PLA/TPU10	3.03	52.40	6.80	0.75	0.86	9.63	34.39	16.65	2.50	–1.31
PLA/TPU15	2.86	44.30	10.75	0.69	0.79	9.13	32.93	20.63	3.22	–2.05
PLA/TPU20	2.64	43.50	21.60	0.62	0.82	8.43	32.77	26.69	4.15	–1.72
PLA/TPU30	2.08	27.50	40.00	0.54	0.83	6.36	28.79	32.04	5.35	–1.62
PLA/TPU40	1.70	20.80	40.00	0.50	0.85	4.61	26.36	32.04	6.02	–1.41
PLA/TPU50	1.32	17.08	40.00	0.38	0.85	2.41	24.65	32.04	8.40	–1.41

**Table 5:** Normalized Output and Deviation Sequence

	normalized data					deviation sequence				
sample name	*E*	*σ*_u_	*ε*_b_	*R*_f3_	*R*_r_	*E*	*σ*_u_	*ε*_b_	*R*_f3_	*R*_r_
PLA/TPU5	1.00	1.00	0.00	0.02	1.00	0.00	0.00	1.00	0.98	0.00
PLA/TPU10	0.89	0.87	0.25	0.00	0.71	0.11	0.13	0.75	1.00	0.29
PLA/TPU15	0.83	0.74	0.44	0.12	0.00	0.17	0.26	0.56	0.88	1.00
PLA/TPU20	0.74	0.73	0.74	0.28	0.31	0.26	0.27	0.26	0.72	0.69
PLA/TPU30	0.49	0.37	1.00	0.48	0.41	0.51	0.63	0.00	0.52	0.59
PLA/TPU40	0.27	0.15	1.00	0.60	0.61	0.73	0.85	0.00	0.40	0.39
PLA/TPU50	0.00	0.00	1.00	1.00	0.61	1.00	1.00	0.00	0.00	0.39

**Table 6:** GRC and GRG Values

	GRC						
sample name	*E*	*σ*_u_	*ε*_b_	*R*_f3_	*R*_r_	GRG	rank
PLA/TPU5	1.00	1.00	0.00	0.34	0.00	0.47	4
PLA/TPU10	0.82	0.80	0.00	0.33	0.00	0.39	7
PLA/TPU15	0.75	0.66	0.47	0.36	0.00	0.45	5
PLA/TPU20	0.66	0.65	0.66	0.41	0.42	0.56	1
PLA/TPU30	0.49	0.00	1.00	0.49	0.46	0.49	3
PLA/TPU40	0.00	0.00	1.00	0.55	0.56	0.42	6
PLA/TPU50	0.00	0.00	1.00	1.00	0.56	0.51	2

## 4 Conclusion

In this study, PLA/TPU SMPCs were successfully prepared with varying TPU content (5, 10, 15, 20, 30, 40, and 50%) using high-speed thermo-kinetic mixing. Comprehensive analyses, including morphological, chemical, thermal, and mechanical characterizations, were conducted using SEM, FTIR, XRD, TGA, DSC, and DMA, as well as tensile and bending tests. SEM analysis confirmed the attainment of homogeneous PLA/TPU mixtures through the high-speed thermo-kinetic mixing approach. This compatibility between PLA and TPU led to a demonstrated decrease in the *T*_g_ in PLA/TPU SMPCs, as verified by DSC and DMA results. Additionally, this enhanced compatibility correlated with an improvement in mechanical properties compared with reports in the literature. Enhancements in mechanical properties were achieved compared to those reported in the literature, indicating the efficacy of the high-speed thermo-kinetic mixing method in comparison to conventional melt blending techniques. Based on the mechanical test results, for the same amount of TPU in the mixture, the mechanical properties of the mixture presented here are much higher than those reported in previous studies, which make the presented mixtures more suitable for practical applications.

Cold programming of the mixtures was systematically examined, and a shape recovery test was done. Results showed that mixtures with TPU content more than 20% are capable of CP, and they have shape recovery more than 80% which makes them useful for practical applications. The mixture with 5, 10 and 15% TPU has better mechanical properties; however, they cracked during the programming at RT which jeopardizes their shape memory properties. Although the addition of TPU increases the flexibility of the mixture and cold programmability, it decreases the mixture’s mechanical properties. Thus, a multiobjective optimization was employed to optimize the blends based on key mechanical properties (tensile modulus, tensile strength, and strain at break) and shape memory characteristics (shape recovery and shape fixity ratio) by using GRA to find the mixture with optimum properties.

Based on the GRA results, the PLA/TPU20 mixture, exhibiting a tensile modulus of 2.64 GPa, tensile strength of 43.50 MPa, strain at break of 21.60%, shape fixity ratio of 0.62, and shape recovery ratio of 0.82, emerged as the optimum sample in the realm of cold-programmed SMPCs. The results of these studies can be used to prepare SMP mixtures with higher mechanical properties and adequate shape recovery for practical applications.
